# Automated analysis of immunoglobulin genes from high-throughput sequencing: life without a template

**DOI:** 10.1186/2043-9113-3-15

**Published:** 2013-08-27

**Authors:** Miri Michaeli, Michal Barak, Lena Hazanov, Hila Noga, Ramit Mehr

**Affiliations:** 1 The Mina & Everard Goodman Faculty of Life Sciences, Bar-Ilan University, Ramat-Gan 52900, Israel

**Keywords:** Immunoglobulin, B cells, High-throughput sequencing, Insertions-deletions, Repertoire, Lineage tree, Somatic hyper-mutation

## Abstract

**Background:**

Immunoglobulin (that is, antibody) and T cell receptor genes are created through somatic gene rearrangement from gene segment libraries. Immunoglobulin genes are further diversified by somatic hypermutation and selection during the immune response. Studying the repertoires of these genes yields valuable insights into immune system function in infections, aging, autoimmune diseases and cancers. The introduction of high throughput sequencing has generated unprecedented amounts of repertoire and mutation data from immunoglobulin genes. However, common analysis programs are not appropriate for pre-processing and analyzing these data due to the lack of a template or reference for the whole gene.

**Results:**

We present here the automated analysis pipeline we created for this purpose, which integrates various software packages of our own development and others’, and demonstrate its performance.

**Conclusions:**

Our analysis pipeline presented here is highly modular, and makes it possible to analyze the data resulting from high-throughput sequencing of immunoglobulin genes, in spite of the lack of a template gene. An executable version of the Automation program (and its source code) is freely available for downloading from our website: http://immsilico2.lnx.biu.ac.il/Software.html.

## Background

### Immunoglobulin (antibody) genes and lymphocyte repertoires

The immune response involves cells of various types, most notably the B and T lymphocytes, which perform the roles of antibody production (B cells), killing virally-infected or transformed cells (cytotoxic T cells), or directing the immune response in many ways (helper T cells). These lymphocytes express a large diversity of receptors called B and T cell receptors (BCR and TCR, respectively), which recognize foreign antigens as well as self-molecules. The genes for BCRs and TCRs are somatically rearranged from segments that are randomly selected from gene segment libraries, with much imprecision in the joining of gene segments [1-4]. T and B cells are formed throughout life; those lymphocytes whose receptors bind their cognate antigen proliferate and perform their effector functions, with some of these cells remaining in the system as long-lived memory cells.

In addition, B cells mutate their receptor genes (also called immunoglobulin genes) during the immune response, and selection processes acting on the mutants result in improved affinity of the BCRs and of their secreted form–i.e., the antibodies–to the antigen. Thus the diverse repertoire of T and B lymphocytes within each individual is constantly changing. While TCR and BCR diversification endows the system with the ability to produce receptors recognizing any possible biological molecule or pathogen, the staggering receptor diversity–up to 10^11^ different B or T cell clones in each human, for example–makes it very difficult to study how the lymphocyte repertoire changes under various conditions. Such studies are very important for, e.g., understanding how the immune system copes with complex infections such as those with the human immunodeficiency virus (HIV) or hepatitis B virus, and finding the best neutralizing antibodies [5]; for elucidating the changes in immune function during natural aging [6]; or for correctly classifying lymphocyte cancers [4].

### High-throughput sequencing of immunoglobulin genes–the challenge

The recent development of high throughput sequencing (HTS) enables researchers to obtain large numbers of sequences from several samples simultaneously. HTS has a great advantage over classical sequencing methods in the field of immunoglobulin (Ig) gene research, as it enables us to extract more sequences per sample and is sensitive enough so we can identify different unique sequences [3,5-8]. HTS has already been available for several years; thus, data cleaning programs have been developed, to perform the identification of molecular identification (MID) tags and primers and discard low-quality sequences (reviewed in [9]). However, the software packages normally used to clean HTS data and identify mutations rely on the existence of a “reference” or “template” for the whole gene, to which all sequences can be compared. Such a template does not and cannot exist for the highly diverse repertoire of Ig genes, and thus the available programs cannot deal with the cleaning of Ig genes, for the following reasons.

First, the large numbers of sequences that are obtained from HTS must be curated, that is, assigned to samples, cleaned from artifact or low quality sequences, and put in the correct orientation. Doing this manually for hundreds of thousands or millions of sequences is obviously not feasible. We have developed a data cleaning program, Ig-HTS-Cleaner, that addresses this need [9]. This program performs the following tasks. First, it assigns the sequences to samples according to their MID tags, and discards sequences in which MID tags cannot be identified at both ends–which is useful in case samples are coded not by a single MID tag but by a combination of MID tags at both ends. It also discards sequences in which the MID tag combination is identifiable but does not appear in the list of sample codes, because these sequences are most probably artifactual chimeric (hybrid) sequences created during PCR amplification or sequencing. Second, Ig-HTS-Cleaner identifies the primers at both ends of each read, using dynamic programming with the user-defined limit to the number of mismatches allowed, in cases where an exact match cannot be found. Primers need to be identified in order to be removed from the read, because mismatches in these segments may be PCR errors and thus should not be counted as bona fide somatic mutations. Third, the program discards all reads that do not conform with the length range of Ig genes (and thus may be irrelevant genes or chimeric sequences), and those that have quality scores below the user-defined threshold. All discarded read are counted and stored in separate files for quality control. This enables the user to study the effects of changing the program’s parameters (such as the maximum number of allowed mismatches in primer search), and thus optimize the parameters for the dataset at hand.

Second, the large number of processing steps required even after data are curated means that analysis of the data manually–even with most programs having the ability to run large batch jobs–would take too long and would be very repetitive and tedious. In the days before HTS, it still took us weeks to analyze data from studies that generated only hundreds of sequences. Thus, as many of these steps as possible needed to be automated. For this to work, the different data formats must be reconciled, such that in each step the program used will be able to work on the output from the previous step. We describe our proposed solution in the Methods section.

### Further processing of immunoglobulin gene data

In order to analyze the Ig gene repertoire and the mutations that have accumulated in these genes, several preliminary steps must be taken above and beyond data cleaning. The component segments of each gene (germline segments) must be identified, sequences should be grouped into clonally-related sets, alignments and lineage tree analysis should be performed in order to infer the junction regions between segments, and then one needs to correctly identify the mutations and their most likely history in each clone. The community of researchers focusing on BCR bioinformatics has developed various software packages to perform this task over the years (reviewed in [10]). The main contribution of our studies to this collection of methods was the introduction of lineage tree analysis. Lineage tree generation is performed using our program IgTree© [11], implementing a distance method-based algorithm that finds the most likely tree with a high probability. After the construction of lineage trees, various mutational analyses that rely on tree structure can be performed, such as amino acid (AA) substitution counts [12-14], which may determine the effect that mutations have on the final antibody, and analysis of the frequencies of replacement and silent mutations (RS analysis) [15,16], which provides insights regarding the nature of selection. Lineage trees also enable the investigation of B cell clonal dynamics, such as initial affinity or selection threshold of clones, by measuring their graphical properties, using our program MTree© [17].

Recently, we have also developed a program, Ig-Indel-Identifier, that deals with the insertions and deletions (indels) near homopolymer tracts–a known problem with the 454 HTS platform, which is more severe in Ig gene analysis due to the lack of template or reference genes. While the two above-mentioned programs are not guaranteed to identify each and every artifactual insertion, deletion or chimeric sequence, they manage to identify many of these cases, so that manual examination of the sequences is only needed in very few cases.

The analysis of an Ig sequence dataset is thus composed of between 10 to 20 different steps, each performed by a different program. As long as B cell repertoire research had been based on Sanger sequencing, yielding at most hundreds of sequences in each study, each of these analysis steps could be performed separately and semi-manually for each clone. With the introduction of HTS, however, the enormous number and diversity of Ig gene sequence reads makes it impossible to manually analyze the sequencing results. To address this challenge, we have developed an almost completely automated analysis pipeline, which integrates the programs used in each step of the analysis and enables us to analyze large numbers of reads. Some of the tools we have developed have the potential to be useful for other situations where a template is lacking. In this paper, we present the structure of this automated pipeline, the programs used in each step, and preliminary results from some of the studies that were performed so far using this pipeline.

## Methods

### Automated analysis pipeline for Ig gene HTS data analysis

**Figure 1:**
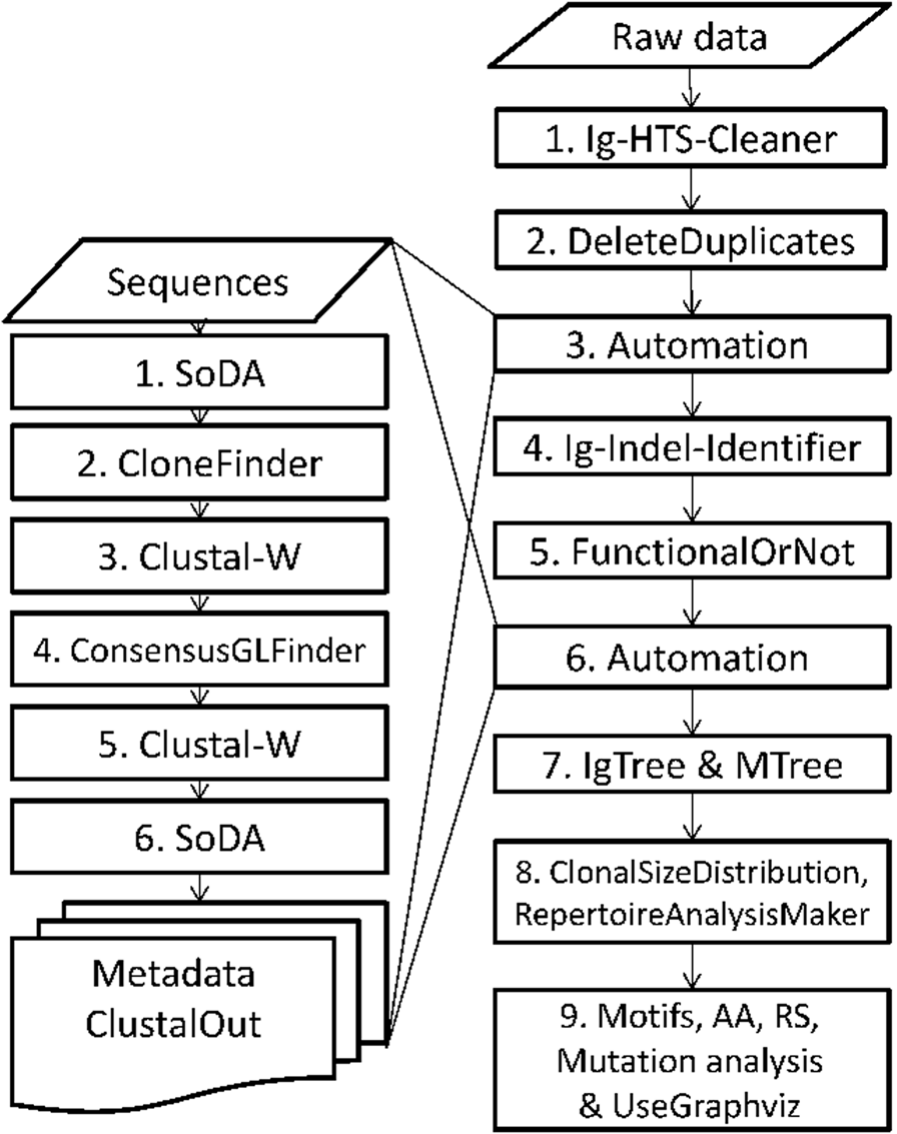
**A scheme of the cleaning and analysis pipeline of high throughput Ig gene sequences.** The process starts with raw data reads from high throughput sequencing (right column). Each rectangle in the right column represents an independent module or program that is used in the analysis pipeline, and thus can be skipped through. The Automation program is represented in the right column as a single step, and the lines coming out of the “Automation” rectangles lead to the left column which presents the Automation program flow, which is constructed of several steps as detailed in the manuscript. The Automation program receives as input a file of sequences, and creates metadata and alignment files for each clone for further analyses. The arrows in the right column represent the recommended flow of the cleaning and analysis process as created and done by the authors. The arrows in the left column represent the flow of the Automation program, as discussed in the manuscript.

We created an almost completely automated pipeline that includes all steps a set of sequences has to pass, starting with the 454 raw data up to the final results of the analysis. Figure 1 describes this pipeline, and the sections below describe the steps that it takes to analyze the data.

### Part one: from raw data to Ig gene sequences ready to be analyzed

Raw data are cleaned and assigned to samples using Ig-HTS-Cleaner, as explained above. The next step is to find the germline segments composing each rearranged gene. Mutated sequences must be compared to the pre-mutation rearranged sequence to identify mutations. Usually, this sequence is not available, so it is reconstructed by identifying the original gene segments used, based on highest homology to the mutated sequence; the germline junction region is then deduced from a consensus of all clonally related sequences. Several programs may be used for identifying the germline segments and the junction regions, such as IMGT/V-QUEST [18], SoDA [19] or iHMMune-align [20]. We currently use SoDA for our analyses as it is most convenient to use.

Next, we discard duplicate sequences, as they may be derived from the PCR reaction so we cannot be sure they represent the actual cell numbers. The routine “DeleteDuplicates” performs the following steps. For each sample, the program creates a file that contains only unique sequences, i.e. sequences that differ from each other by insertions, deletions or substitutions. For each unique sequence, the program lists all its duplicate sequences, if any, in a separate file.

Finally, each sample is analyzed by our “Automation program”, which consists of several steps (see below).

### Part two: clone assignment and alignment–the Automation program

The “Automation program” is a UNIX based program, written in C++ and Java, and designed to process Ig sequences from B cells whether they come from HTS or not. The purpose of the Automation program is to automate the complex processing and analysis of large numbers of Ig gene sequences including grouping them into clones, creating lineage trees using IgTree© [11], and analyzing all the mutations using MTree© and the additional programs described below. All these steps have existed in the group as separate programs prior to the automation. Manual analysis, as was executed until now, is out of the question due to the much larger numbers of sequences obtained by HTS. The Automation program makes this analysis very fast, easy and accurate. An executable version of the Automation program (and its source code) is freely available for downloading from http://immsilico2.lnx.biu.ac.il/Software.html. In order to run the Automation program, one should have a UNIX-based operating system, and only a basic knowledge in UNIX commands is required (all requirements and instructions for running the Automation program are delivered with the executable files).

During an analysis run, the Automation program runs the sequences in a local version of SoDA to identify the V(D)J segments of each sequence and its germline (GL) sequence. Then, it sorts the sequences into groups (clones) having the same V(D)J segments. All GL sequences in each clone are aligned using a local version of ClustalW2 [21], under the basic assumption that all the sequences in a clone have developed from the same GL ancestor. Therefore, we use the alignment to find the consensus GL sequence, which is the common ancestor of all sequences within the clone, and is combined of the identified GL segments and the majority consensus for the junction regions (N-nucleotides). After finding the consensus GL sequence, all the sequences in a clone and the consensus GL sequence are aligned again. (The alignment provides several parameters and a pir file, which are later used for IgTree© and tree drawings). The next step is applying the local version of SoDA to the GL sequence in order to obtain more parameters regarding the clone, such as regions of CDRs and FRs, and a summary of all clone parameters is written into a file called ‘Metadata’.

Following the first run of the Automation program, we proceed to clean artifactual insertions/deletions from the sequences using Ig-Indel-identifier, as explained above. We then perform a functionality analysis on the files containing sequences without artifact indels, which divides the sequences of each sample into functional, non-functional and indeterminate sequences according to SoDA definitions. Non-functional sequences often have a frame-shift or a stop codon in their sequence. Indeterminate sequences usually contain short J segments, such that the reading frame cannot be identified by SoDA. Thus, the routine FunctionalOrNot creates three files using the Metadata files from the automation process, containing functional, indeterminate and non-functional sequences, respectively. In addition, it lists the number of functional, indeterminate and non-functional sequences in each clone. The user may proceed with the analysis using only the functional sequences, only non-functional sequences, or all sequences. After these intermediate steps, we run the automation on the cleaned files, finally receiving groups of cleaned, clonally-related and aligned sequences, ready for lineage tree analysis.

### Part three: finally, repertoire, lineage tree and mutation analyses

During the clonal expansion of B cells in response to antigen, Ig gene sequences accumulate mutations via somatic hypermutation and thus diversify. An easy way to track and analyze the relationships between clonally related Ig gene sequences is by using lineage trees. The tree root is the ancestor sequence, usually the rearranged, pre-mutation sequence. Each tree node represents a single mutation (point mutation, insertion or deletion).

IgTree© and MTree© are run on the aligned clonally-related groups of sequences. IgTree© produces the tree files as adjacency lists, which serve as the input for MTree; tree drawing files from which one may create the actual drawings using a graphics programs, e.g. Graphviz; and input for the various mutation analysis programs. Our routine “UseGraphviz” runs Graphviz, a program for lineage tree drawings, on all tree files automatically.

We then perform repertoire analysis (that is, enumerate the clones and sequences that are based on each combination of the V,D and J germline segments) and clonal size distribution analysis, and analyses of mutation targeting motifs, AA substitution, RS and lineage tree measurements. HTS provides us with many more sequences than previously, allowing deeper observations into the BCR repertoire in various clinical conditions. Many researchers, who have performed HTS on Ig gene sequences so far, focused mainly on repertoire analysis (e.g., [6,22]). Therefore, we can compare our repertoire analysis results to those of previous studies, even though the first Ig gene HTS studies were published only about four years ago [7]. In order to analyze repertoires, we created a table of all possible V and J combinations. For each sample, we enumerate the clones and unique sequences that use each V-J combination. After the tables are created by the RepertoireAnalysisMaker script we calculate the average percent of the frequency of clones and unique sequences of each V-J combination, across all individuals within the same group. This normalizes the cases where some samples contain more sequences and/or clones than others due to PCR bias. Clonal size distribution analysis examines the distribution of the numbers of sequences in all clones in a sample. To create clonal size distributions for all samples, we wrote a script (ClonalDistribution) that creates a tab-delimited .txt file, containing the number of sequences and unique sequences for each clone. The results can then be graphed using any graphics program, such as Microsoft Excel.

## Results and discussion

### Example: Ig gene sequences from human lymph nodes

We demonstrate the performance of our pipeline by presenting the analysis of Ig gene sequences from 15 human lymph nodes (LNs) (from a study that will be published elsewhere; the purpose of this example is only to demonstrate how we use the analysis pipeline). Human DNA aliquots extracted from these 15 samples were subject to Ig gene amplification by PCR and the products were sequenced on the Roche 454 FLX Titanium platform. An Ig-HTS-Cleaner run on ~527,000 reads took approximately 5 minutes on our UNIX server, which is equipped with 16GB RAM. Out of the ~527,000 reads, 63,283 reads contained MID tags at both ends of the read. In the next step, Ig-HTS-Cleaner discarded 2,029 reads that did not contain identifiable primers at both ends. All LN reads were in the desirable length range. Ig-HTS-Cleaner discarded 707 sequences that did not pass the average quality threshold, which we set to be 25. Finally, when Ig-HTS-Cleaner had finished running, we were left with a total of 60,547 remaining sequences unambiguously assigned to human LN samples. Next, for each of the 15 samples we discarded duplicate sequences, ending up with a total of 25,733 sequences. We then ran the automation process on each of the new files received in the previous step. Each run took a few hours.

We ran Ig-Indel-Identifier on the 25,733 sequences from the 15 samples. Out of these sequences, 17,763 sequences did not contain indels at all; this is reasonable, since somatic hypermutation inserts mostly single base substitutions [23,24]. On the other hand, 7,970 sequences contained indels. Applying Ig-Indel-Identifier on each of the 15 LN samples took approximately 5 minutes to run on an Intel ® core ™2 CPU 6700, 2GB RAM 2.67GHz.

We then ran the automation process again on each of the files containing only sequences without indels, received after cleaning with Ig-Indel-Identifier. This time, each run took less time because the files contained fewer sequences, as sequences with artifact indels were already discarded.

**Figure 2:**
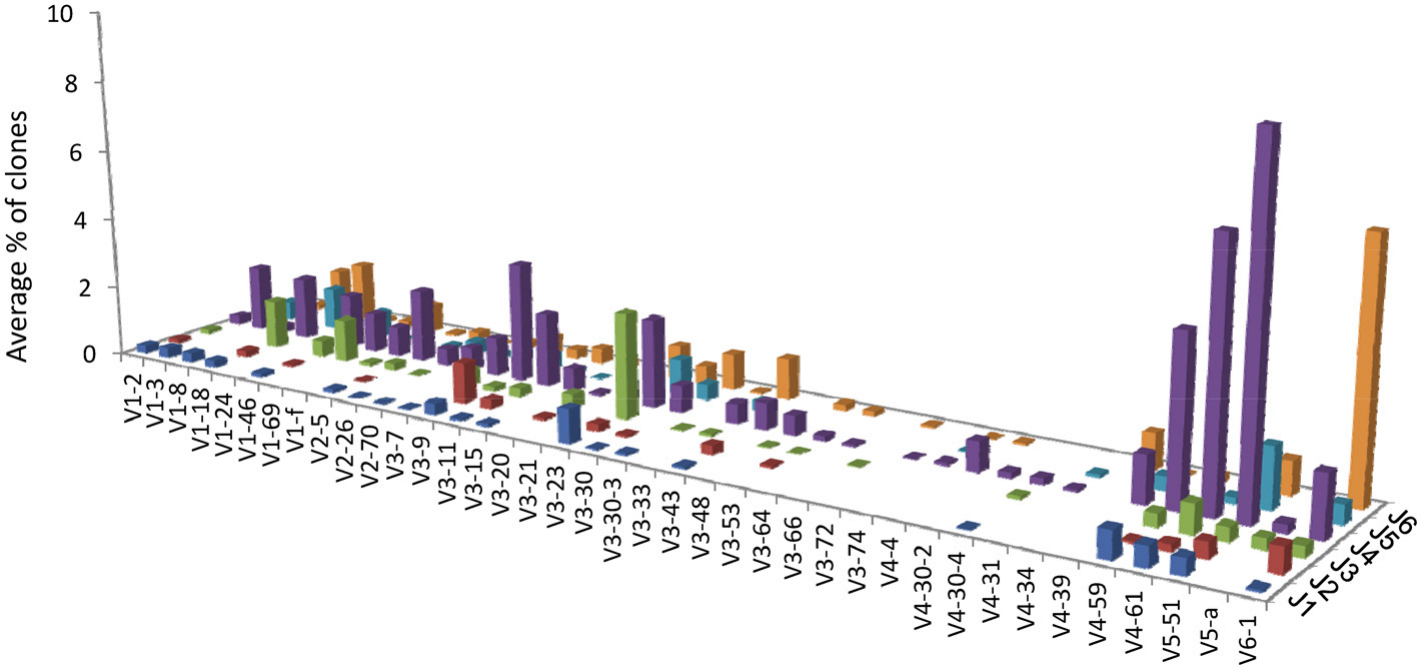
**Average percentages of clones in each VH-JH combination, in human LNs.** This graph represents one of the possible outcomes of our proposed pipeline, which, in this case, contributes to Ig gene repertoire diversity analyses.

We then performed a functionality analysis and got 10,029 functional sequences, one indeterminate sequence and 7,733 non-functional sequences. We proceeded with the analyses with both functional and non-functional sequences from each sample, as functionality is only deduced from frame shifts and stop codons, which could be sequencing artifacts; and performed repertoire and clonal size distribution analyses, as described above. Other research groups may be interested in analyzing only functional (or non-functional) sequences, so the functionality analysis should be performed before the second Automation program run. Figure 2 presents the repertoire of average percentage of the number of clones in each V-J combination found in the 15 human LN samples. Thus, the example demonstrates the use of our analysis pipeline. The performance of the individual programs used by the pipeline has already been discussed in the original publications.

## Conclusions

In summary, the analysis pipeline presented above makes it possible to analyze the data resulting from HTS of Ig genes, in spite of the lack of a template gene. Our pipeline is highly modular. Each of the stages of our pipeline can be run separately and does not depend on any other program in the automation process. In addition, the above-described automation process can be modified to contain or discard specific stages, and can easily be changed to include different orders of steps or even new steps.

## Competing interests

The authors declare that they have no competing interests.

## Authors’ contributions

MM created Ig-HTS-Cleaner, Ig-Indel-Identifier and more scripts mentioned in the manuscript, performed the data analyses and performance review, and drafted the manuscript. MB created IgTree© and participated in the creation and integration of the Automation program. LH and HN participated in the creation and integration of the Automation program. RM supervised the creation of the automated pipeline and the analyses performed, and finalized the manuscript. All authors read and approved the final manuscript.
